# Task sharing of a psychological intervention for maternal depression in Khayelitsha, South Africa: study protocol for a randomized controlled trial

**DOI:** 10.1186/1745-6215-15-457

**Published:** 2014-11-21

**Authors:** Crick Lund, Marguerite Schneider, Thandi Davies, Memory Nyatsanza, Simone Honikman, Arvin Bhana, Judith Bass, Paul Bolton, Michael Dewey, John Joska, Ashraf Kagee, Landon Myer, Inge Petersen, Martin Prince, Dan J Stein, Graham Thornicroft, Mark Tomlinson, Atalay Alem, Ezra Susser

**Affiliations:** Alan J Flisher Centre for Public Mental Health, Department of Psychiatry and Mental Health, University of Cape Town, 46 Sawkins Road, Rondebosch, Cape Town, South Africa; Perinatal Mental Health Project, Alan J Flisher Centre for Public Mental Health, Department of Psychiatry and Mental Health, University of Cape Town, 46 Sawkins Road, Rondebosch, Cape Town, South Africa; School of Applied Human Sciences, University of KwaZulu-Natal, Howard College Campus, Durban, 4000 South Africa; Department of Mental Health, Johns Hopkins Bloomberg School of Public Health, 703 Hampton House, 624 N. Broadway, Baltimore, MD 21205 USA; Center for Refugee and Disaster Response, Departments of International Health and Mental Health, Johns Hopkins Bloomberg School of Public Health, 703 Hampton House, 624 N. Broadway, Baltimore, MD 21205 USA; Health Service and Population Research Department P060, Institute of Psychiatry, Psychology and Neuroscience, King’s College London De Crespigny Park, London, SE5 8AF UK; Department of Psychiatry and Mental Health, University of Cape Town, J2 Groote Schuur Hospital, Observatory, 7925 Cape Town, South Africa; Alan J Flisher Centre for Public Mental Health, Department of Psychology, Stellenbosch University, Private Bag X1 Matieland, 7602 Stellenbosch, South Africa; School of Public Health and Family Medicine, University of Cape Town, Room 5.51 Falmouth Building, Observatory, 7700 Cape Town, South Africa; MRC Unit on Anxiety and Stress Disorders, Medical Research Council, PO Box 19070, Tygerberg, 7505, Cape Town, South Africa; Department of Psychiatry, Faculty of Medicine, Addis Ababa University, P.O. Box 9086, Addis Ababa, Ethiopia; Mailman School of Public Health, Columbia University, 722 West 168th Street Room 1508, New York, NY 10032 USA; New York State Psychiatric Institute, 1051 Riverside Drive, New York, NY 10032 USA

**Keywords:** Community health workers, Cost-effectiveness, Counseling, Hamilton depression rating scale, Maternal depression, Randomized controlled trial, Task sharing

## Abstract

**Background:**

Maternal depression carries a major public health burden for mothers and their infants, yet there is a substantial treatment gap for this condition in low-resourced regions such as sub-Saharan Africa. To address this treatment gap, the strategy of “task sharing” has been proposed, involving the delivery of interventions by non-specialist health workers trained and supervised by specialists in routine healthcare delivery systems. Several psychological interventions have shown benefit in treating maternal depression, but few have been rigorously evaluated using a task sharing approach. The proposed trial will be the first randomised controlled trial (RCT) evaluating a task sharing model of delivering care for women with maternal depression in sub-Saharan Africa. The objective of this RCT is to determine the effectiveness and cost-effectiveness of a task sharing counseling intervention for maternal depression in South Africa.

**Methods/Design:**

The study is an individual-level two-arm RCT. A total of 420 depressed pregnant women will be recruited from two ante-natal clinics in a low-income township area of Cape Town, using the Edinburgh Postnatal Depression Scale to screen for depression; 210 women will be randomly allocated to each of the intervention and control arms. The intervention group will be given six sessions of basic counseling over a period of 3 to 4 months, provided by trained community health workers (CHW)s. The control group will receive three monthly phone calls from a CHW trained to conduct phone calls but not basic counseling. The primary outcome measure is the 17-Item Hamilton Depression Rating Scale (HDRS-17). The outcome measures will be applied at the baseline assessment, and at three follow-up points: 1 month before delivery, and 3 and 12 months after delivery. The primary analysis will be by intention-to-treat and secondary analyses will be on a per protocol population. The primary outcome measure will be analyzed using linear regression adjusting for baseline symptom severity measured using the HDRS-17.

**Discussion:**

The findings of this trial can provide policy makers with evidence regarding the effectiveness and cost-effectiveness of structured psychological interventions for maternal depression delivered by appropriately trained and supervised non-specialist CHWs in sub-Saharan Africa.

**Trial registration:**

Clinical Trials (ClinicalTrials.gov): NCT01977326, registered on 24/10/2013; Pan African Clinical Trials Registry (http://www.pactr.org): PACTR201403000676264, registered on 11/10/2013.

## Background

### Significance of task sharing to primary health care in sub-Saharan Africa

Mental disorders represent a substantial and growing proportion of the burden of disease in sub-Saharan Africa, in part because of demographic and epidemiological transitions [1]. In South Africa, approximately 16.5% of adults have had a Diagnostic and Statistical Manual of Mental Disorders (Version 4) disorder in the past year, including 4.9% with major depression [2], and neuropsychiatric disorders are ranked as the third most disabling set of conditions [3]. Mental disorders are associated with substantial lost income and days out of role in South Africa [4, 5]. Mental disorders also predict a number of other negative health outcomes that are highly relevant to the disease burden in sub-Saharan Africa, for example increasing risk for HIV transmission and reducing the likelihood of adherence to anti-retroviral treatment [6]. However, despite the overwhelming need for mental health services, only 50% of African countries have a mental health policy and 70% of countries spend less than 1% of their health budgets on mental health [7, 8]. Psychiatric hospitals remain the dominant mental health resource, with many hospital beds used for forensic or custodial care [8–13]. The gap between the number of people who require and those who receive treatment (also known as the “treatment gap”) is substantial, and is estimated to be 75% in South Africa [14]. It is unlikely that this treatment gap will be bridged by mental health specialists. In 2010, there were approximately one psychiatrist per 2 million people, one nurse working in mental health care per 164,000 people, and one psychologist per 2.5 million people in Africa [8]. Centralized services and a critical shortage of specialist mental health workers are the main causes of the large treatment gap for mental disorders in sub-Saharan Africa [15]. Geographical inequities, the historical legacy of colonial and apartheid health systems, and a shortage of mental health specialists underpin this treatment gap in South Africa [16].

To address this gap, the strategy of “task shifting” or “task sharing” has been proposed by the World Health Organization and a number of leading international researchers [17, 18]. Task sharing has been defined as the delivery of low cost interventions by non-specialist health workers, who are trained and supervised by mental health specialists, through routine health care delivery systems [19–21]. Task sharing has been distinguished from task shifting in that the former involves an emphasis on the role of supervisors in “sharing” the task of caring for the clients. Task sharing can reduce the pressure on psychiatric specialists, and enable packages of mental health care to be delivered to a larger number of people within a given population. The South African government’s National Mental Health Policy Framework (2013–2020) proposes substantial investment in mental health services, with an important role for primary care and community health workers (CHWs) [22]. Some pilot studies have shown that CHWs can be trained to successfully provide task sharing interventions for depression in South Africa, with structured and supportive supervision [23, 24].

### Burden and significance of maternal depression

Within the mental health field, maternal depression presents a major public health concern [25]. Prenatally, studies have shown that anxiety and depression lead to increased risk factors for mothers and children, including shorter gestation periods, compromised fetal neurodevelopment, and lower birth weight [25]. The persistence of maternal depression postnatally has also been found to negatively affect the quality of childcare, child safety, and appropriate response by mothers to their children, resulting in impaired cognitive, social, emotional, and behavioral development in the child [26–28].

The prevalence of depression amongst perinatal mothers in low- and middle-income countries (LMICs) is estimated to be between 15% and 57% [29–32]. Studies in Khayelitsha have reported the point prevalence of depression in pregnant women as high as 39% [33], and in postpartum women as 34.7% [34]. Perinatal depression in this setting is exacerbated by low socio-economic status, unemployment, violence, crime, HIV status, poor health care, poor emotional and practical support from partners, social isolation, and interpersonal disputes [34].

While maternal and child health have been identified as key priorities for intervention by the South African Department of Health [35], no programs for the treatment of maternal depression have as yet been introduced in a systematic manner within the public health sector [36]. Poor delivery of mental health services is exacerbated by large workloads and insufficient mental health training for primary health care workers [37].

To our knowledge, there have been no randomized controlled trials (RCTs) in sub-Saharan Africa to address maternal depression with community health workers through a task sharing approach, and there are limited research data in Africa to address key questions regarding the delivery of such interventions [38]. This is partly reflective of limited capacity in African countries to design and execute research that addresses these questions [39].

### Potential interventions for maternal depression

There are several effective and promising psychosocial and psychological treatments for maternal depression [40], with growing evidence from LMICs suggesting that low cost, task sharing approaches to improving mental disabilities can contribute to improving quality of life and reduce disabilities [41, 42].

Problem solving therapy (PST) has been found to effectively address depression and other common mental disorders, and is particularly effective within a task sharing approach due to its easily understood structure and cost effective nature [37, 42, 43]. Preliminary studies in Harare, Zimbabwe, and in Cape Town and KwaZulu-Natal, South Africa, found evidence that locally adapted PST, delivered by CHWs, is effective in reducing symptoms of depression and other common mental disorders [24, 37, 42]. PST is seen as a beneficial form of therapy within LMICs because many common mental disorders are rooted in everyday social and health problems [42, 43]. PST seeks to address this through collaborative identification and exploration of problems, and identification and implementation of solutions. Moreover, it is brief, structured, and focused on the present, with an active collaboration between patient and counselor [43]. In addition, cognitive behavior therapy also has a strong international evidence base for treating adult depression [44], including maternal depression in Pakistan [45], and has shown promise in the context of task sharing in local pilot studies in Cape Town and KwaZulu-Natal [24].

In LMICs, there have been very few RCTs evaluating delivery of care for persons with mental disorders in primary health care [46], with previous RCTs largely focused on stand-alone interventions of specific medications or psychological therapy [20].

### Objectives

The overall objective of the trial is to determine the acceptability, effectiveness, cost-effectiveness, and potential sustainability of task sharing mental health care for maternal depression compared to enhanced usual care in South Africa.

The specific objectives are:

1. To determine the effectiveness and cost-effectiveness of a task sharing psychological intervention delivered by CHWs, compared to enhanced usual care in South Africa, on both primary outcome measures (of effectiveness and cost-effectiveness) and on a series of secondary outcome measures.
2. To examine factors influencing the implementation of the task sharing intervention and potential for future scale up by assessing acceptability, feasibility, sustainability, quality, and safety.
3. To adapt and evaluate locally relevant but generalizable measures, including both symptom severity measures and functioning measures, for evaluating the effectiveness and cost-effectiveness of task sharing care for maternal depression.

The primary hypothesis of the trial is that mothers with depression who receive the task-shared care will have improved clinical outcomes, as defined by a 40% reduction in depressive symptoms measured on the Hamilton Depression Rating Scale (HDRS) at 3 months postnatally, compared to mothers who receive enhanced usual care.

## Methods/Design

### Trial design

The study design is an individual-level RCT.

### Setting

The study site will be the periurban settlement of Khayelitsha, on the outskirts of Cape Town, South Africa. Khayelitsha is one of the fastest growing township settlements in South Africa, and representative of many other periurban settlements in the country. The area has one district hospital to serve a population of approximately 500,000 people, with five Community Health Centres and eight day clinics [47]. Most people live in overcrowded, poor living conditions, with high levels of unemployment and poverty. Many houses or shacks are without electricity, running water, or an indoor sewerage system [48]. Since the introduction of democracy in 1994, efforts have been made to improve dwelling conditions and many low cost houses have been built in Khayelitsha. Many residents have moved to Cape Town from rural areas in the country, and the predominant language is isiXhosa.

### Participants

Participants will be drawn from the isiXhosa language group in South Africa, who reflect the majority of the black African population in the Western Cape, and who were historically marginalized from services under the apartheid era, with these inequities persisting into the present. The participants will be women 18 years or older screened for depression using the Edinburgh Postnatal Depression Scale (EPDS) at their first antenatal clinic visit, typically in the second trimester.

Eligibility criteria:

- Women attending one of two antenatal clinics (Michael Mapongwana Community Health Center (CHC) or Site B CHC) in Khayelitsha, presenting for their first antenatal visit, no later than 26 weeks gestation.
- Living in Khayelitsha.
- 18 years or older.
- Screen positive for depression with a cut off of 13 or more on the EPDS.
- Able to give informed consent.
- Speak isiXhosa as a first language (or competently as a second language).
- Do not require urgent medical attention or have severe mental health problems, defined as a diagnosis of schizophrenia, bipolar mood disorder, or currently experiencing an episode of psychosis.

### Recruitment and screening

**Figure 1:**
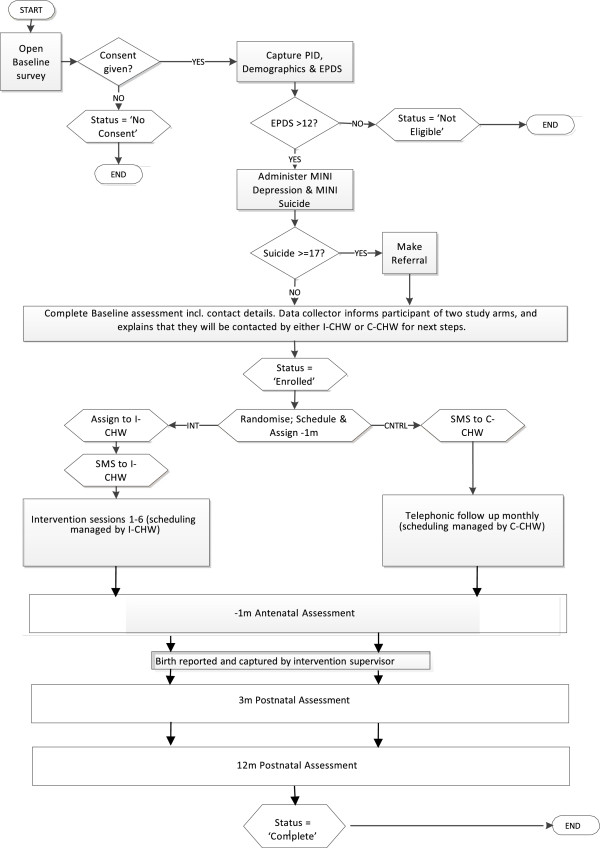
**Africa focus on intervention research for mental health (AFFIRM) flow chart.** PID, Participant identification number; EPDS, Edinburgh postnatal depression scale; MINI, Mini International Neuropsychiatric Interview; I-CHW, Intervention arm community health worker; C-CHW, Control arm community health worker; −1 m, 1-month antenatal follow-up assessment; SMS, Short messaging service text message.

Potential participants will be recruited by trained field workers at the routine screening of pregnant mothers during their first antenatal visit at the two CHC Maternity and Obstetric Units in Khayelitsha. This is the point at which pregnant women are most likely to attend, typically in their first or second trimester, and therefore presents a real-world screening opportunity if this intervention is to be scaled up by the national Department of Health. Approximately 92% of women attend antenatal clinic checkups in South Africa [49]. The limitations of this approach include the possibility that, since women are routinely tested with rapid HIV tests at their first antenatal visit, detected rates of depression may be conflated with mothers who are acutely distressed after discovering that they are HIV positive for the first time on the day of the interview.Screening by the field workers will be performed in collaboration with the nurses conducting their checkups in order to ensure that all potential participants attending the clinics on the day are screened. There will be no advertisements for the project. Women who give informed consent to the field worker will be recruited through a three-stage screening process: i) verbal confirmation of the eligibility criteria for the study; ii) completion of a set of demographic questions; and iii) administration of the EPDS. Participants who score 13 or above on the EPDS will be recruited into the study and asked to complete the full baseline assessment with the field worker (Figure 1). The baseline assessment will include basic demographic questions regarding age, education, employment status, income, household composition, housing, and receipt of social grants.

If a screened participant shows signs of suicidality (scoring 17 or higher on the Suicidality module of the Mini International Neuropsychiatric Interview 6.0.0) she will be referred to the psychiatric nursing sisters at the CHCs and, if necessary, these nurses will refer her to the local Khayelitsha District Hospital for further management, including access to antidepressant medication if indicated.

### Randomization and treatment allocation

Following the baseline assessment and recruitment by the field workers, participants will be randomly allocated to the psychological intervention or enhanced usual care control condition by the computerized data management system.

#### Sequence generation and allocation concealment

A random number list will be generated by the data management system with individual numbers automatically allocated to each participant. The investigators and staff do not have access to this list or allocation system. After allocation by the system, a text message will be sent to one of the six intervention arm CHW counselors (for those women allocated to the intervention) using a round robin approach for the six CHW counselors, or to the two control CHWs conducting the phone calls for the control (enhanced usual care) arm. When the field worker completes the baseline assessment, she will inform the participant that they will either receive an appointment to meet the CHW for an initial counseling session at the clinic, or that they will receive a phone call from a CHW to check on their progress. Further descriptions of the intervention and enhanced usual care conditions are provided below.

#### Blinding

Given the nature of the study intervention and outcome, blinding is a particular concern. Investigators will only have access to anonymized baseline data at the end of the recruitment phase. Data will be presented for Group A and B without the investigators knowing what A and B refer to in terms of intervention and control arms. Data analysis will be blinded until the analysis is finalized and approved by all investigators. In addition, all assessments will be conducted by field workers who will be blinded to patient treatment and will work independently of the CHWs. Participants will not be informed of the study hypothesis. CHWs involved in the study will be trained in the importance of adhering to the study protocol and random checks will be undertaken by the project manager within the experimental and control arms to minimize contamination. Field workers will not have meetings or interactions with the CHW counselors to discuss progress of the trial while the trial is being conducted. Each team (CHW counselors and field workers) will be managed by a different team leader. The project field supervisors (research officer and specialist mental health counselor), and CHWs are therefore the only project members who are unblinded.

### Interventions

#### Intervention arm

##### Personnel: recruitment, training, and supervision

Twelve CHWs currently employed by a local non-governmental organization (NGO) will be trained to deliver the manual-based counseling intervention. CHWs will receive 5 days of training in basic counseling and the intervention for maternal depression from the Africa Focus on Intervention Research for Mental Health mental health counselor (a Clinical Social Worker). Six of the 12 CHWs will then be chosen at the end of the 5-day training to implement the intervention. Selection will be based on CHWs’ motivation to continue with the intervention, their understanding of the course materials, and their empathy and interpersonal style, as displayed during mock counseling sessions conducted during the training.

CHWs will receive weekly group-based specialist mental health support and supervision from a specialist mental health counselor (Social Worker). This will include i) supervision, including case reviews, discussion of difficult cases, developing supportive relationships with general health providers and trouble-shooting, and ii) consultation with the specialist mental health counselor regarding the management of an emergency (such as suicidal behavior in a study participant). In addition, the specialist mental health counselor will also meet with the CHWs individually on a monthly basis to discuss progress with their work. These consultations, referral, and supervision processes are intended to provide a pragmatic model for the task sharing intervention that can be scaled up in other similar settings.

To protect the CHWs against burnout, a procedure will be put in place as part of the initial training whereby CHWs will firstly be taken through a program of recognizing stress reactions or burnout symptoms in themselves. Second, during the weekly meetings, the CHWs will be encouraged to talk through difficult situations – not only to manage the situations better but also to help the CHWs to recognize their own feelings about the case. The mental health specialist will in turn go through a similar process of support with the project manager. If required, CHWs will be provided with free counselling sessions from a professional counselor or psychotherapist not involved in the study.

##### Participants: counseling intervention

Participants randomized to the intervention arm will receive the structured manual-based psychological treatment by the trained CHWs, who are known and accepted within the local community. They will receive six counseling sessions (of approximately 1 hour in duration each), which include aspects of psycho-education, PST, behavioral activation, cognitive reframing (healthy thinking), and relaxation training. This has been informed by a review of the available literature, and formative qualitative research conducted in the 12 months preceding the trial, which indicated the need for problem solving skills, extending the range of social support and usual activities through behavioral activation, and cognitive reframing to address patterns of persistent negative cognitions. The intervention was also informed by similar manuals developed in the field and consultation with co-investigators.

The counseling sessions will be conducted approximately every 2 weeks at the clinic or at the participant’s home (by prior arrangement). Counseling sessions will be scheduled to align as closely as possible with routine antenatal check-ups at the clinic. All counseling sessions will be initiated antenatally, but if the mother is enrolled in the study late in her pregnancy or delivers her baby early, the sessions may continue into the postnatal period, until all six sessions are completed. As part of the intervention, CHWs have been trained to follow-up women with maternal depression who miss sessions and encourage them to participate in the counselling sessions. This follow-up will include phone calls to the woman if a session is missed. During the phone call counselors will explore reasons for missing the session and help the woman to overcome barriers that may be preventing her from attending. A drop-out will be defined as a woman who misses three consecutive counselling sessions.

##### Fidelity of intervention

Fidelity of the intervention is essential to ensure that the counselling sessions are conducted competently by the CHW counselors and according to the manual-based protocol. The assessment of fidelity will be performed using a series of measures:

- The CHW counselors will complete a checklist for each session, indicating what was covered during the session.
- The specialist mental health counselor will observe initial sessions and discuss these sessions with the CHWs.
- Group supervision sessions will be used to revise the CHW counselors’ knowledge of key aspects of the intervention manual at regular intervals during the trial.
- All counseling sessions will be audio recorded and the mental health specialist will listen to all initial sessions and then a random selection of subsequent sessions.

#### Control arm

##### Personnel: recruitment, training, and supervision

Two independent CHWs will be trained by the mental health counselor to conduct phone calls to the mothers in the control arm every month for three months. The CHWs will be trained to identify suicidal ideation and refer mothers to psychiatric nurses if needed. They will not be trained in any of the counselling techniques used in the intervention arm.

##### Participants: control intervention

Participants randomized to the control group will receive enhanced usual care. Usual care involves attendance at antenatal and postnatal clinics on a voluntary basis, where the focus is on fetal and infant physical development and immunization. Enhanced usual care will comprise, in addition to usual care, regular monthly telephone calls over 3 months, delivered by two CHWs trained in conducting structured phone calls but not trained in counseling techniques. The telephone calls by the CHWs will engage participants in a brief conversation about i) how the woman is feeling (general health question); ii) whether there are any major changes in the woman’s life and, if so, what these are (including starting any medication); iii) whether she has someone to assist her if she needs help; iv) whether she or her baby have been visited by any community workers from other organizations and description of these visits; and v) whether there is suicidal ideation. If there are signs of major problems (e.g., abuse or other social difficulties), participants will be provided with contact details of relevant health services and NGOs. If there are signs of suicidal ideation, they will be referred to the psychiatric nurses at the clinic. Similarly to the intervention arm, unless their health or behavior makes it impossible for them to continue to receive the telephone calls or participate in follow-up assessments, suicidal participants will be retained in the study.

In determining the optimal number and frequency of phone calls to the women in the control arm, a balance needs to be retained between the need to monitor any adverse changes in the women’s mood, and not being too intrusive or providing too intensive an intervention that would not represent usual care. Following formative research and discussion with the research team, it was decided that monthly telephone calls given over 3 months (in keeping with the timing of the counseling intervention) are sufficiently frequent to monitor any major changes in mood, offer referrals, and provide information about services and thereby constitute enhanced usual care.

##### Fidelity of the control arm

Fidelity will be assessed by the mental health supervisor who will monitor the calls randomly for 1 hour per CHW per week. In addition, the CHW who conducts the calls will be required to complete a checklist, setting out the information covered during the call, any need for referrals, and adverse events.

### Number and duration of visits

Participants in both arms will be screened at baseline and receive follow-up assessments at 1 month antenatally and 3 and 12 months postnatally. Participants in the intervention arm will be asked to attend six counselling sessions, and a sub-sample (n = 36) will be randomly selected to participate in a semi-structured qualitative interview after their 3 month postnatal assessment. The assessments, counselling sessions, and semi-structured interview will each take approximately one hour. A sub-sample (n = 10) of the control arm participants will be randomly selected to participate in a semi-structured qualitative interview after their 3 month postnatal assessment. All participants will be involved in the study for approximately 15 to 18 months (including follow-up assessments).

### Outcomes

#### Outcome measures

The primary outcome measure is depression, assessed using the HDRS 17-item version [50]. Secondary outcome measures will be assessed using the following instruments:

- Edinburgh Postnatal Depression Scale (EPDS) [51].
- Mini International Neuropsychiatric Interview 6.0.0 [52]: Major Depressive Episode and Suicidality modules.
- A Health Care Utilization Questionnaire, adapted from the Client Socio-Demographic and Service Receipt Inventory – European Version [53], and the Client Service Receipt Inventory [54].
- World Health Organization Disability Assessment Schedule 2.0 [55, 56].
- Cape Town Functional Assessment Instrument for Maternal Depression; this instrument has been developed during the formative preparation of the trial, and uses the method for developing a local culturally valid functional assessment instrument developed by Bolton et al. [57].
- Household Food Insecurity Assessment scale [58].
- Multidimensional Scale of Perceived Social Support [59, 60].
- Alcohol Use Disorders Identification Test [61].
- Brief drug, abuse, and HIV questions: this involves a few questions regarding any drug use, or experience of physical or sexual violence; in addition, participants will be asked to disclose their HIV status.
- Obstetric and child outcome measures at 3 and 12 month postnatal follow-up assessments. These will include height and weight of the infant, head circumference, adherence to child immunization (marked on the baby’s Road to Health Chart), and prevalence of diarrheal disease and respiratory tract infections.

The above outcome measures will be administered at baseline, at 1 month antenatally, and at 3 months and 12 months postnatally. To our knowledge, participants will not be participating in any other research study, and we will include questions about the receipt of other interventions in all baseline and follow-up interviews.

All assessments will be conducted by trained field workers using handheld electronic devices. This will allow data to be uploaded to a central database in real time, minimize the likelihood of errors at data collection or capturing stages, and will facilitate the continuous checking of data quality by the research officer.

All instruments have been translated into isiXhosa. Some instruments have been used in a number of studies with isiXhosa-speaking participants. The other instruments have been forward and back-translated, and their performance carefully reviewed during the pilot phase.

### Sample size and power calculation

The sample size of 420 women (210 in each arm) is based on the following assumptions: two-sided testing at alpha = 0.05, beta = 0.1 (i.e., 90% power), and a 30 to 50% reduction in depressive symptoms at 3 months postnatally as measured by the HDRS-17 in the control arm based on enhanced usual care. Absolute effect sizes of interest range from 20 to 40%. Based on previous cohort studies and trials in mental health care in this community, we feel that 10% attrition over the study period is realistic. In addition, we conservatively estimate contamination between the trial arms of the order of 5% (that is, 5% of women in the control arm will experience the treatment effect of the intervention through exposure to women in the intervention arm). Based on these assumptions, we estimate that 420 women in the trial will be required to detect an intervention effect of 20% or greater between the two arms.

The sample of 420 women makes provision for subjects that might move away or refuse to take part in the study at a later stage. If subjects in the study drop out or are unable to continue participating in the trial they will not be replaced. Drop out from care is defined as three consecutive missed appointments, for either the counselling sessions or telephone calls. We will endeavor to continue to collect data from people who move out of the area provided they give informed consent to continued participation, and can be contacted. After a participant has missed five consecutive follow-up assessment appointments for a specific assessment point, they will be deemed to have dropped out of the study.

### Statistical methods

A data management and statistical analysis plan has been developed during the preparations leading up to the trial. This details the procedures for data quality assurance and quality control (including standard operating procedures), data entry, management and cleaning, and data analysis (including *a priori* comparisons, interim analyses, and Data Safety and Monitoring Board (DSMB) instructions).

Data will be entered at the time of interview via handheld devices (cell phones) and automatically stored in a secure firewall-protected database. Data analysis will take place using the latest version of STATA (College Station, Texas, USA). All analyses will be blinded until the analysis is finalized and approved by all investigators. Data cleaning based on frequency distributions and logic checks will follow standard procedures with reference to source documents as required. Exploratory analyses will include frequency distributions and measures of central tendency and dispersion, as appropriate, with 95% confidence intervals. Throughout these analyses, stratifications will be by participant age, and depression severity at enrollment. Throughout, bivariate comparisons will employ χ^2^, Fisher’s exact, student’s T- or rank-sum tests, as appropriate; all statistical tests will be two-sided at α = 0.05. The primary analysis will be by intention-to-treat, and secondary analyses will be on a per protocol population, defined as women who complete all sessions in either arm of the trial. The primary outcome measure will be analyzed using linear regression adjusting for baseline symptom severity measured using the HDRS-17. The validity of regression assumptions will be checked using residual plots. Logistic regression will be used to model some secondary outcomes, which are measured on a binary scale.

In relation to the economic analysis i) costs associated with delivering the task sharing intervention, as well as the consequential impact on service use, will be analyzed using multiple regression analysis; bootstrap methods will be used if the residuals of the regression model are non-normally distributed. ii) Further regression models will include the main clinical measures so that the relationship between costs and outcomes can be assessed.

### Ethical considerations

Ethical approval for research with human subjects was obtained from the University of Cape Town Human Research Ethics Committee (Ref no 226/2011) and the National Institute of Mental Health (NIMH) DSMB prior to the start of recruitment. Each participant is providing informed consent prior to participation in the trial. On the whole, the trial will pose minimal risks to the participants. In order to minimize risk, careful training and supervision of the counselors and field workers will occur. A monitoring system is in place for adverse and serious adverse events, with a protocol for the management of these events and reporting at appropriate times to the DSMB. Participants will be asked about suicidal ideation at all contacts (including intervention and control sessions and all baseline and follow-up assessments), and if participants report suicidal ideation, a protocol is in place for referral to the psychiatric nurses at the clinic. Unless their health or behavior makes it impossible for them to continue to receive counseling sessions or participate in follow-up assessments, suicidal participants will be retained in the study. Uptake of psychiatric services by these participants will be monitored for both groups.

Quality assessment of the study will occur at a number of levels:

- The mental health counselor will oversee the training, supervision, and support of the CHW counselors and CHWs telephoning the control group participants. They will hold regular individual and group supervision sessions to review sessions completed and issues arising.
- Further refresher training will be provided if this is deemed necessary at any given point in time.
- The research officer will train, supervise, and support the assessment team in the baseline and follow-up assessments.
- All data from the baseline and follow-up assessments and reports from sessions (e.g., dates, length of session, manual topic covered) will be captured using mobile technology. The database will be reviewed on a continuous basis by unblinded members of the team to check on quality.
- The trial statisticians will be asked to review the data at key points during the life of the trial.

Reports will be submitted to the NIMH DSMB twice per year for external control of quality. All serious adverse events will be reported to the NIMH DSMB within 48 hours of being reported to the trial team.

It is expected that participants will experience a number of direct benefits as a result of receiving care in the intervention arm of the trial. These may include improved mood, reduction of symptoms, improved social functioning, stronger social support networks, improved bonding with infant, improved health care utilization by mother and infant, and the development of problem solving skills. In addition to the above direct benefits, the study is likely to yield generalizable knowledge about the outcomes of task sharing counseling interventions for maternal depression. This may inform the development and scaling up of such interventions in routine practice in South Africa and other African countries.

## Discussion

In summary, there is a substantial treatment gap for mental disorders in sub-Saharan Africa, and addressing this treatment gap requires innovative public health strategies, such as task sharing. Maternal depression is a key public health concern, with high prevalence rates reported among deprived communities in the Western Cape and KwaZulu-Natal provinces in South Africa. Maternal depression has negative health consequences for both mothers and their infants. There are a number of potential psychological interventions that have shown benefits in the treatment of maternal depression, but very few have been rigorously evaluated using a task sharing approach in sub-Saharan Africa.

Research to date indicates the need for a RCT to evaluate the effectiveness and cost effectiveness of a task sharing intervention for maternal depression in a low income context, such as Khayelitsha, South Africa. This intervention needs to be contextualized to local needs and tailored for specific disorders. The general principles of task sharing need to be documented in such a way that they identify the common ingredients of effective approaches for mental health services across the sub-Saharan African region. The proposed trial will be the first RCT evaluating task sharing models of delivering care for persons with maternal depression in sub-Saharan Africa.

## Trial status

The trial is currently in the recruiting stage. Data collection began in October 2013, and will continue until October 2014.

## Abbreviations

CHC: Community health center
CHW: Community health worker
EPDS: Edinburgh postnatal depression scale
DSMB: Data safety and monitoring board (USA)
HDRS: Hamilton depression rating scale
LMICs: Low and middle income countries
NGO: Non-governmental organization
NIMH: National Institute of Mental Health (USA)
PST: Problem solving therapy
RCT: Randomized controlled trial.
